# Advancing health equity in Nova Scotia by exploring gaps in healthcare delivery: a mixed methods protocol

**DOI:** 10.1007/s43999-025-00062-4

**Published:** 2025-04-24

**Authors:** Jennifer Lane, Neda Alizadeh, Christine Cassidy, Neil Forbes, Holly McCulloch, Katrina Jarvis, Helen Wong, Courtney Pennell, Lori Wozney, Kris Lane, Brittany Barber, Kelly Lackie, Bukola Oladimeji, S. M. Kawser Zafor Prince, Drew Burchell, Noah Doucette, Cyril O’Brien, Wyatt LeRoy, Kendra MacEachern, Elizabeth Obeng Nkrumah, Joshua Edward, Arezoo Mojbafan, Megan White, Tatianna Beresford, Janet Curran, JianLi Wang, Marilyn Macdonald

**Affiliations:** 1 School of Nursing, Faculty of Health, Dalhousie University, 5869 University Ave, Box 15000, Halifax, NS B3H 4R2 Canada; 2 Aligning Health Needs and Evidence for Transformative Change: A Joanna, Briggs Centre of Excellence, Dalhousie University, Halifax, NS Canada; 3 Education, Research, and Applied Studies, Indigenous Knowledge, University of Prince Edward Island, Prince Edward Island, Charlottetown, Canada; 4 IWK Health, Halifax, NS Canada; 5 Faculty of Management, Dalhousie University, Halifax, NS Canada; 6 Faculty of Medicine, Dalhousie University, Halifax, NS Canada; 7 Nova Scotia Health, Halifax, NS Canada; 8 Tajikeimɨk, Millbrook, Canada; 9 Faculty of Health, Dalhousie University, Halifax, Canada

**Keywords:** Health equity, Structural determinants of health, Intersectionality Theory, Mixed methods research, Regional health services, Sex and gender-based analysis plus

## Abstract

Population health issues are addressed by various regional initiatives in the Canadian province of Nova Scotia (NS). A need for research on the root causes of health inequities suggests there may be a lack of evidence to inform current initiatives within the region. To address this gap, a three-phase sequential mixed methods study called *Advancing Health Equity in NS by Exploring Gaps in Healthcare Delivery* will operationalize Intersectionality Theory and employ an integrated knowledge translation approach to identify and explore gaps in health service delivery. This will promote a better understanding of how to improve the integration of health equity in health service and delivery systems and thus population health and well-being. The following objectives will be addressed in each phase: 1) create an inventory of NS-relevant knowledge that relates to health equity, 2) examine the integration of health equity in NS health service and delivery systems using a context-specific health equity lens, and 3) mobilize knowledge on how gaps in service delivery can be addressed to improve the integration of health equity and better meet the needs of people living in NS. The study results from this protocol will be used to integrate health equity in NS health service and delivery systems, enhancing the quality of care for populations rendered vulnerable by structural inequalities, and working to prevent negative impacts to health and wellbeing.

**Supplementary Information:**

The online version contains supplementary material available at 10.1007/s43999-025-00062-4.

## Background

The urgent need for healthcare reforms globally, nationally, and locally [1–3] is rooted in the distribution of resources and influenced by intersecting health equity-related indicators (HEI) (e.g., age, appearance, beliefs, belonging, citizenship, disability, ethnicity, gender, geography, race, sex, sexual orientation, and socioeconomic class) [4–25]. This intersectionality is tied to complex systems that need to be comprehensively examined before they undergo change. Otherwise, existing health disparities and marginalization of equity-denied groups could be furthered within healthcare systems [26]. The concept of intersectionality emerges from Black feminist scholarship and can be used to explore the organization of power in economic, ecological, and political contexts, which shape outcomes, including those that are health related [12, 27]. Intersectionality permits the use of identity, and the politics thereof, to examine contextual operations of power, systems of privilege, interlocking oppressions that sustain social inequalities [12, 28, 29], and to elucidate transformational opportunities for bringing about equitable change. We will do this by generating intersectional analyses that explore the delivery of health services within a sufficiently diverse population [27].

### Regional diversity and healthcare delivery in Nova Scotia

In Canada, regional diversity exists within and between populations, geographies, healthcare system governance structures, service delivery models managed by provincial/territorial health authorities, and levels of internet access [30–34]. Nova Scotia (NS) is one of ten Canadian provinces [34]; there is significant diversity and local knowledge that can be leveraged to improve and strengthen the healthcare system [1, 35, 36]. There are 13 Mi'kmaq communities in NS; [37, 38]; Indigenous Peoples living in the province are a distinct part of the general population (e.g., First Nations including Mi’kmaq, Inuit, and Métis) [39]. A growing proportion of the Mi’kmaw population resides in Halifax, the province’s Capital city [40]. There are also over 50 historical African Nova Scotian communities (i.e., foundational Black Nova Scotians who came to the region over 400 years ago) [41]. Additionally, there are record numbers of recently landed immigrants [42], increasing numbers of visible minority groups [43], the highest proportions of gender diverse individuals aged 15–34 (170% higher than the national average) [44] and disabled people (30.4%) compared to other regions in Canada [45], a rural population of 40% [46], and 22.2% of Nova Scotians are aged 65 or older [47]. The province is also home to sexual minorities (e.g., lesbian, gay, bisexual) [48], intersex individuals [49], and people with Acadian and Gaelic [50] heritage, among others. Notably, these groups do not operate separately from one another. Individuals can simultaneously belong to more than one group, with group memberships potentially shifting over time, making population health interventions particularly challenging to sustain if they are not systemically scaled [51–54].

Various regional initiatives aim to address the healthcare needs of diverse populations in NS, but few are controlled by the groups they aim to serve, or are operating independently from the colonial health system governed by the provincial government. Exceptions to this include the Mi’kmaw health and wellness organization, called Tajikeimɨk [38], as well as the Wije’winen Health Centre situated in the Mi’kmaw Native Friendship Centre that provides culturally relevant care to urban Indigenous Peoples living in Halifax Regional Municipality. Organizations established by and for people of African descent include the NS Association of Black Social Workers (since 1979) [55] and Health Association of African Canadians (since 2000) [56].

Regional initiatives that aim to address the healthcare needs of diverse populations in NS, but are part of the provincially funded health system include the NS Brotherhood and Sisterhood, who work to improve the overall health and wellbeing of people of African Descent by increasing access to health care in the community [57, 58]; the Newcomer Health Clinic, which is an initiative that provides both preventative and primary health services for refugees in the greater Halifax area [59]; and PrideHealth, which aims to improve access to safe, coordinated, and comprehensive primary health care for members of the Two Spirit, lesbian, gay, bisexual, trans, queer, intersex, and asexual (2SLGBTQIA +) community [60]. The 2SLGBTQIA + acronym is one of many acronyms that are used to describe a group of communities, obscuring intersections of race, ethnicity, disability, and other social categories of identity [29]. This acronym is being used here because this is the term that PrideHealth uses to describe the population they aim to serve, and a single community is referred to for the same reason. Two Spirit is a term that is specific to Indigenous Peoples in North America who are often diverse in terms of their gender and/or sexual orientation and hold historically significant community-specific roles [61]. The “ + ” is meant to represent anyone who is a member of this community but does not identify with the previous terms.

In addition to being part of the provincially funded health system, the Brotherhood, Sisterhood, PrideHealth, and Newcomer Clinic operate out of the same provincial health authority management zone, close to the provincial capital [62], and may not be accessible to those living in the other three zones [63]. If a person is outside the service area, then the allocation and operationalization of healthcare services and resources across urban and rural communities present additional challenges [64]. A person who is eligible to access more than one of these services might have to prioritize one aspect of their identity over another when accessing health care. People experiencing intersectional inequalities who are ineligible for these services would have to access care within a system that uses a one-size-fits-all approach to healthcare delivery. People who are disabled, low-income, have a history of abuse, and/or any number of additional factors that impact how they experience health care (but are not addressed by existing approaches to healthcare delivery) are thus forced to access a system that is not designed equitably. This is not to say that initiatives serving equity-denied groups are a bad thing or that the provincial health authority is somehow wrong to try to address the underservicing of these groups. Rather, it may just be that approaches serving specific groups are more effective when they are operated and controlled by those groups, outside of systems where the unequal distribution of power has yet to be addressed. A more effective approach could involve a model of care that integrates health equity across NS health service and delivery systems [65].

### Equity-advancing policy change in Nova Scotia

The need to advance equity in NS is recognized in the *Dismantling Racism and Hate Act* passed by the NS Government in 2022 [66], and is meant to support initiatives like the 2023 *Equity and Anti-Racism Strategy*, which outlines the province’s plan to address health and social inequities [35]. Although efforts to address health inequities in Canada are increasing, implications of structural forces shaping access to health-related resources often fail to address intersectional inequalities, leaving underlying power dynamics unchallenged [9, 11, 12, 17]. Characterizing the implications of systemic inequities that uniquely impact equity-denied groups in relation to different aspects of the healthcare delivery process may be a way to identify gaps in healthcare delivery that emerge from power imbalances. Yet, the focus remains on groups who are denied equity instead of the unjust systems where inequities are rooted even though an ineffective health system is an issue that impacts everyone living in the region [65]. Notably, as of Oct, 2024, there were over 145,000 people living in NS without a primary care provider, a number that was being viewed with cautious optimism at the time, having decreased from almost 165,000 since the previous month [67].

A current shift in policy is in response to an urgent need to improve access to equitable care for all people living in NS [1, 68]. To promote equitable healthcare delivery close to home, by providers who are knowledgeable about the health needs of the diverse NS population, fulsome understandings of regional factors influencing health and wellbeing are required to appreciate how power imbalances are inextricably linked to health disparities [9, 10, 12, 52, 54, 69, 70]. The COVID-19 pandemic laid bare pre-existing systemic issues, furthered gaps and resource deficiencies, and exposed the need to be inclusive of all groups and individuals [13, 71, 72]. To address this urgent need, gaps in healthcare delivery should be identified and explored using contextually relevant HEI to inform a model of care that is designed to *equitably* serve the diverse social identities, geographic locations, and healthcare needs of *all* people living in the region.

### Aim and objectives

Project ADDING HEAT (**AD[D]**vanc**ING HEA**lth equi**T**y in NS by Exploring Gaps in Healthcare Delivery) is a three-phase sequential mixed methods study [73] that will examine NS health service and delivery systems to better meet the diverse needs of people living in the province. Recommendations will be made on how care delivery processes, provider organization, and service management can be more equitable. This study will address the primary research question: *How can health equity be advanced in NS by identifying and exploring gaps in healthcare delivery?* Each phase has its own objectives:

**Phase 1 Objective:** Create an inventory of NS-relevant knowledge that relates to health equity by carrying out the following activities:

o organize activities that engage equity-denied groups, record information on the engagement process, and obtain informal feedback on the project from attendees, so unmet health needs can be used to identify areas for improvement within NS delivery systems; and
o conduct a scoping review of international standards of care, NS clinical practice guidelines, and NS policies that healthcare providers can use to deliver more equitable care to people living in NS.

**Phase 2 Objective:** Examine the integration of health equity in NS health service and delivery systems by exploring:

o the wellbeing of NS inhabitants and their perceptions of care delivery to identify opportunities to better meet their health needs and improve access to health services; and
o barriers and facilitators to coordinating inclusive, people-centred, and quality health care in NS.

**Phase 3 Objective:** Translate study findings into recommendations on how care delivery processes, provider organization, and service management can be more equitable, facilitate their uptake by end-users, and inform future research on advancing health equity in NS that will address gaps in healthcare delivery identified and explored in this study.

## Methods

Community partnerships are a critical resource in this study and strategically engaging members of equity-denied groups is a means by which the inherent power imbalances that exist between researchers and research participants will be addressed. Community partnerships will be vital to answering the “how” in our research question as the project unfolds. The methods below outline the approach that will be used to identify and explore gaps in healthcare delivery and how partnerships will be formed. What was conceptualized at the time of publication is thus outlined in what follows and that which has yet to unfold will be guided by the Knowledge-to-Action-Ethics Cycle as described [73].

### Setting

The research will be conducted in NS, which sits on the unceded and ancestral territory of the Mi’kmaq People. We will engage NS partners (members of various groups, communities, and professions, including end users) from diverse practice settings and communities.

#### Involving Indigenous Peoples in general population research

To demonstrate respect for and understanding of their unique status in Canada [74–76], efforts will be made to meaningfully recognize Indigenous Peoples as part of the general NS population. This work will be overseen by a working group and include exploring data governance and stewardship to ensure knowledge is used ethically and in beneficial ways [75, 77]. Regular meetings will be held, and a group of Indigenous team members (NF, CP, WL, MW) will be supported by the non-Indigenous Principal Investigator (JL) in scrutinizing study methods and promoting culturally appropriate community engagement practices. Specific attention will be given to engaging Indigenous communities in ways that adhere to local customs and practices [78]. This work will begin by leveraging existing relationships for the purpose of engaging Indigenous communities, coalitions, and/or organizations to determine level of interest in the study (if any). The appropriate authorities will be engaged as the need to do so is identified by the working group. For example, data governance will need to be addressed if Indigenous Peoples participate in the study because data collected from them belongs to them [75]. This will require us to consult with community leaders on how we should approach this process, even though the study is not specifically involving an Indigenous community. We do not yet know the degree to which Indigenous Peoples will share their data with us, and until that has been determined, we cannot explore data governance. Implications of this include uncertainty around exactly how statistical analyses will be employed because decisions about data that belongs to Indigenous Peoples cannot be made without them [78].

We will meet with members of communities to which people on our team belong and explore the promotion of our study, but a different trajectory may be required when engaging in such activities with Indigenous team members. This is because it takes additional time to build trust with those who have been affected by colonization as Indigenous Peoples have in Canada, affirm respect Indigenous community customs, and identify mutual benefits in researcher-community relations [78]. The relationships that Indigenous members of the working group have with individuals from their own communities exist outside the context of this study and as such, negotiation around project goals may be required. Agreements may also require ongoing negotiation (much like the informed consent process) as the project unfolds and will be explored at individual and community levels. For example, individual governance agreements will be offered to Indigenous participants who express an interest in being interviewed. Members of the working group developed an individual governance agreement template, which has been appended (Appendix 1). On Reserve Tribal Council engagement will involve requesting to meet with Chief and Council.

Mi’kmaw Ethics Watch (MEW), an REB whose purpose is to protect Mi’kmaw cultural knowledge [79], will be informed about the project on an ongoing basis. This will promote Indigenous Peoples’ right to self-determination and data sovereignty, ensuring that MEW decides whether the study needs to be reviewed [78], aligning with the United Nations Declaration on Rights of Indigenous Peoples, specifically that of participating in decision-making and right to self-representation [80]. The rights of Indigenous people involved in this study will be promoted in other ways as the project unfolds, according to the direction of Indigenous team members and partners.

### Design

This study will employ a three-phased sequential mixed methods design [73] using an integrated knowledge translation (IKT) approach [81], informed by the operationalization of Intersectionality Theory, community engagement, and the Knowledge-to-Action-Ethics Cycle.

#### Operationalizing Intersectionality Theory

Intersectionality Theory roots structures of inequality in historical and ongoing events, uses the complexity of identity to explore domains of power operating across multiple forms of oppression, and serves to understand health outcomes as a function of unequal access to resources [9, 10, 12, 25, 52, 69, 70, 82–88]. Due to the scale by which domains of power operate, a structural determinants of health framework will be employed to capture social, political, ecological, legal, moral, and commercial factors that influence health outcomes [89]. The structural determinants of health framework is also advantageous to this study’s aims because it complements approaches that can be used to build capacity among healthcare professionals to address structural inequalities in practice [90], thus aligning with practical ways to promote the integration and advancement of health equity across NS health service and delivery systems, and province more generally. We will operationalize this philosophical perspective by adopting social categories of identity as analytic tools that will inform all aspects of the project, including the analysis and presentation of data extracted in the scoping review, collection of sociodemographic data, and sampling in quantitative and qualitative arms of the study (described in more detail later). Initial identity categories will be defined by selecting appropriate subcategories and will become what we are referring to as HEI. As such, a sex and gender-based analysis plus will be generated to examine the integration of health equity in NS health service and delivery systems.

#### Engaging community

By leveraging existing relationships of members of the research team who belong to different equity-denied groups, various NS communities will be engaged. Engaging all groups and communities across the province is not possible. For this reason, we will engage equity-denied groups to which members of our team belong as we search for sources of local knowledge that can be leveraged to improve and strengthen the healthcare system. Those experiencing the most barriers to care are likely to have gathered significant knowledge about what needs to change within health service and delivery systems, and may stand to benefit the most from this study. Engagement efforts will be equitable so the involvement of populations who are often hard to reach due to historical and ongoing discrimination, mistreatment, oppression, and social exclusion is promoted [91, 92]. Moreover, this will ensure people who belong to equity-denied groups have the opportunity to give us feedback on the study as it unfolds to ensure it accurately represents their experiences and perspectives [36]. Leveraging existing relationships will help us cultivate culturally safe interactions through shared experiences, locations, and/or interests. We will build trust via collaborative and respectful practices to explore the involvement of equity-denied NS groups and individuals in this study so we can consult with them on how the data we collect on gaps in healthcare delivery can be used to advance health equity in NS and incorporate this into the recommendations that come out of this study. There are various roles available to individuals who belong to equity-denied groups to facilitate this process that include research team members, recruitment partners, research participants, and community engagement event attendees. This builds community engagement into the project because members of various equity denied groups are involved in every aspect of the research process, many of which can be routinely consulted on decisions that need to be made.

#### Knowledge-to-Action-Ethics Cycle

The Knowledge-to-Action-Ethics Cycle (KTA-E Cycle) is a conceptual framework that facilitates ethical creation, translation, uptake, and application of knowledge by end users [93–95]. The KTA-E Cycle will support our IKT approach and provide a systematic method to examine how knowledge on gaps in healthcare delivery can be translated for uptake by end users and applied to advance the integration of health equity in NS health service and delivery systems. Further, it will be used to guide all consensus-building activities (i.e., initial and follow-up community engagement efforts in phases one and two respectively and full-team consensus meetings at the end of phases one and two), thus facilitating an ethical, evidence-informed approach to advancing health equity in NS.

The KTA-E Cycle was entered by identifying the inadequacy of existing strategies in addressing health inequities and acknowledging that pre-existing knowledge belonging to various groups, particularly those who are equity-denied, needs to be taken up into the health system to address gaps in healthcare delivery that hinder the advancement of health equity in NS. The KTA-E Cycle is comprised of two action phases that are non-linear and can occur simultaneously [93]. First is the knowledge creation phase, which has eleven activities. Knowledge translation activities were initiated from the project’s onset, but will continue to be reviewed, selected, and adapted with community partners throughout the study to improve the contextualization and application of knowledge as well as the sustainability of its use [93]. The second is the knowledge-to-action phase, which has eight activities. Knowledge translation activities were initiated from the project’s onset, but will continue to be reviewed, selected, and adapted with community partners throughout the study to improve the contextualization and application of knowledge as well as the sustainability of its use [93]. Table 1 outlines what has/will be done for each phase and step. Activities will be expanded upon and the original framework adapted as necessary, as the study unfolds. This adaptation will be outlined in a forthcoming publication (see knowledge mobilization section for additional information on dissemination activities).

**Table 1:** Actions informed by the KTA-E Cycle at the time of protocol submission

KTA-E Phase	Step^a^	Activities
Knowledge Creation	Establish partnerships	Relationships with individuals from equity-denied groups were established overtime, prior to the initiation of the project. Partnerships were developed between the PI (a disabled lesbian) and individuals who agreed to join the research team when it formed. This was done by obtaining letters of support from people who would join the team as partners (*n* = 13), which is the team role that is primarily responsible for engaging individuals from equity-denied groups
	Form research question	The PI developed a research question that all members of the team were asked to provide feedback on. Its broad nature is intentional so all people living in NS can participate
	Design project	The PI developed the initial project design, all members of the team were asked to provide feedback, and changes were made accordingly. Phase one community engagement solicited feedback from individuals belonging to equity-denied groups on aspects of the project design that could be changed, including guidance on how Intersectionality Theory would be operationalized
	Seek funding	Grant applications were developed to sustain the project and funding was subsequently awarded. A grant application was under review at the time of protocol submission
	Obtain REB approval	Regulatory compliance was achieved by obtaining research ethics board approval from Dalhousie University. A separate ethics application was submitted to Mi’kmaw Ethics Watch to determine if they needed to review our study at that time (which they did not but this may change so updates are provided in an ongoing manner)
	Recruit participants	Existing relationships between people on the team and members of communities to which they belong are being leveraged as traditional recruitment strategies are also used to mitigate selection bias. Partnerships will continue to be formed to gain support for the study in whatever way partners request that are feasible. Assistance with recruitment will be requested of all partners so members of communities they are from/serve are more likely to participate in the study. Recruitment had yet to be started upon initial submission of this protocol
	Collect data	Data collection had yet to begin upon initial submission of this protocol
	Analyze data	Data analysis had yet to begin upon initial submission of this protocol
	Draw conclusions	Conclusions had yet to be drawn upon initial submission of this protocol
	Publish results	Results had yet to be published upon initial submission of this protocol
	Towards KT and further research	Future research had yet to be conceptualized upon initial submission of this protocol
Knowledge Translation	Review and select knowledge	Knowledge will be reviewed and selected in various ways, including literature reviews, phase one community engagement activities, the scoping review, and will also involve contextualizing preliminary findings with assistance from members of equity-denied groups and other interested partners
	Adapt knowledge to context	Knowledge obtained from members of equity-denied groups in the form of feedback (phase one community engagement) was adapted to the context of the study. Phase two community engagement will involve contextualizing preliminary findings to the lived contexts of individuals who participate in this part of the project
	Access knowledge; identify barriers and supports	The scoping review, survey, and interviews will access knowledge that allows us to identify barriers and supports to integrating health equity across the NS health system
	Apply knowledge	Through community and partner engagement, we will learn how to apply knowledge. Knowledge exchange events will be held at the end of phase two with knowledge users in various roles within the health system, postsecondary education, government, and non-profit organizations to gain insight into how knowledge can be applied by end-users
	Monitor knowledge use	Data governance agreements and guidelines (not yet developed) will be how knowledge use will be monitored
	Evaluate impact of knowledge	Strategies have yet to be developed and will depend on what knowledge is obtained
	Sustain knowledge use	Strategies have yet to be developed and will depend on what knowledge is obtained
	Towards future research and/or continued KT	Findings will inform future research and/or continued KT

^a^Some language has been modified for conciseness or clarity

### Phase 1: create an inventory of NS-relevant knowledge that relates to health equity

#### Community engagement activities

**Fig. 1:**
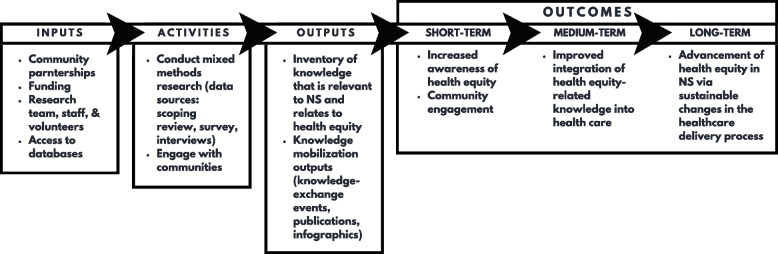
Project ADDING HEAT Logic model

The engagement of Indigenous, African Nova Scotian, 2SLGBTQ, and disabled communities, as well as youth and Arabic-speaking immigrants will be organized in phase one, per the direction of the research team member leading the work to promote a culturally safe approach. Lead organizers will engage members of their communities, including sending invitations to individuals of their choosing. Presentations will be developed and tailored to each group’s interests as they are understood by the lead organizers. We will solicit feedback on the project, including how we plan to operationalize Intersectionality Theory, which will inform the development of an instrument that we are referring to as a “(context-specific) health equity lens”. We will do this by asking people to respond to a set of sociodemographic questions that were developed from initially adopted social categories of identity. People will be asked to provide feedback on whether all important aspects of their identity and factors influencing access to health care were addressed as well as if the language used is respectful, culturally appropriate, and easy to understand (Figure 1).

There will be no formal data collection during these activities because this will undermine the trust-building process. However, information on the engagement process will be recorded and added to the inventory. Additionally, bullet point notes will be taken on what was discussed. These notes will be used to build consensus [91] in which categories and subcategories are adopted to create a context-specific health equity lens that will inform data analysis, inform data triangulation, and promote the integration of different data sources. In accordance with inclusive and equitable community engagement methods [92], the format for each community networking event will be different and informed by community-specific practices, as they are understood by the lead organizers, all of whom will be members of the communities that are being engaged. Community engagement events will also intend to promote the recruitment of research participants; leveraging social networks begins with the research team but will extend beyond existing and new relationships.

#### Scoping review

International standards of care and NS clinical practice guidelines and policies that guide healthcare providers in NS to deliver more equitable care will be identified in a scoping review. We will answer the following review question: What standards of care, clinical practice guidelines, and policies guide health care providers to deliver more equitable care to adult populations in NS, Canada? Answering this question will help us identify gaps in existing knowledge that create barriers to integrating health equity across NS health service and delivery systems. The search strategy will be developed with a librarian scientist (Maritime SPOR Support Unit), registered with the Centre for Open Science (https://osf.io/87kn6/), and carried out in accordance with JBI methodology. English academic and grey literature sources will be considered. All included documents must be about the NS population (> 16 years of age), in a healthcare setting, and explicitly report on at least one HEI. Due to the broad nature of the scoping review and the rapid proliferation of health equity-related initiatives in the last ten years, only sources published from Jan 2014 to the present will be considered.

MEDLINE All (Ovid) and Embase (Elsevier) databases will be used to identify academic sources, and grey literature sources will be identified using an incognito Google search. Two independent reviewers will assess each source against inclusion criteria using the Covidence data synthesis platform. A data extraction tool will be developed and piloted by the research team; key characteristics of sources will be extracted, including information about HEI, which will be categorized using the context-specific health equity lens. Data will be presented in tabular form and synthesized narratively.

### Phase two: mixed methods examination of the integration of health equity in NS health service and delivery systems

A survey and interviews will be conducted to explore aspects of the healthcare delivery process. Quantitative and qualitative data will undergo separate analyses that are later described in more detail. The health equity lens will be used across both data sets to explore gaps and strengths in the healthcare delivery process as well as met and unmet health needs. This is how the integration of health equity in NS health service and delivery systems will be examined.

#### Participants and sample size

Anyone who is 16 years of age or older, lives in NS, accesses the NS health system for care, and understands English is eligible to participate in phase two of Project ADDING HEAT. Based on the sample size calculation, a minimum of 2,395 participants will need to complete the survey, considering the population size (*N* = 823,055 for Nova Scotians 16 + years old) [96], a margin of error of 0.02, and a 95% confidence level [97]. For interviews, we will purposively select a sample of 60 individuals (30 for individual and focus group interviews each) from those who express an interest in participating in an interview. Sociodemographic data collected via the study website (heresearch.ca) will be used to inform purposive sampling. Maximum variation will be achieved across HEI to create as many points of comparison as possible for the purpose of generating an intersectional analysis [85, 98, 99]. This is an adequate sample size for generating an intersectional analysis of qualitative data [86, 99–101] that will promote the exploration of health equity-related gaps in service delivery. While understanding English is required to participate in the study, interpreter services will be offered to prospective participants when they express an interest in participating in an interview or focus group. This intends to promote culturally safe spaces for collecting qualitative data by providing additional support to those who feel they might need help communicating in English.

#### Recruitment

We are not targeting specific groups of people living in NS or communities therein; recruitment strategies will aim to target everyone who meets the eligibility criteria. However, in accordance with principles of equity, diversity, and inclusion [102] we are committed to strengthen engagement with individuals who belong to and/or work with equity-denied groups for the purpose of recruiting diversely. We are positioned to promote the recruitment of a diverse sample population because the majority of our team is comprised of individuals belonging to equity-denied groups. We will do so by distributing recruitment materials to our existing professional and personal networks, per a recruitment strategy that will be co-created during the full-team consensus meeting held at the end of phase one. In addition to this, various recruitment strategies will be used to mitigate selection bias, including promoting the study via partnerships with organizations working with people living in NS, flexible participation options, placing posters in public spaces, and digital marketing. Recruitment ads will include a QR code linking participants to the study website where additional information will be accessible. Once people consent to the collection of their sociodemographic data and/or participating in the survey, they will be given the option to choose between participating in the survey and/or an interview (one-on-one or focus group).

#### Consent

Consent will be informed, ongoing, and dependent on what data are being collected. Consent will be obtained from prospective participants prior to collecting contact information, asking in which aspect(s) of the study the person wishes to participate (i.e. survey and/or interviews/focus groups), and administration of sociodemographic questions. There will be two layers of consent—one for the collection of sociodemographic and survey data, and a second layer for those participating in interviews/focus groups.

#### Survey: data collection and analysis

The survey will include two sets of questions. The first will be the 26-item World Health Organization Quality of Life Scale (WHOQOL-BREF) to examine respondents’ overall wellbeing [103] and identify met and unmet needs of the NS population. The second set of questions will examine various aspects of the healthcare delivery process, identifying gaps (and strengths) that will be further explored (see next paragraph for description of analysis methods). Respondents will be asked to choose their primary role in the health system from one of two options: health service user (HSU) or healthcare worker (HCW). Those who select HSU will be administered the 17-item Patient Experience of Integrated Care Scale (PEICS) that measures patients’ experiences of integrated care [104]. The 21-item Provider and Staff Perceptions of Integrated Care Survey (PSPICS) will be administered to HCW respondents and explore dimensions of care coordination and patient-centeredness from the perspectives of providers and staff [105].

Sociodemographic data will be analyzed using descriptive statistics to describe the sample population. Sociodemographic data are being collected with questions that were developed using the health equity lens and as such, HEI will be created and serve as stratifying variables that are used to explore met and unmet needs of the NS population in relation to gaps (and strengths) in the healthcare delivery process. Linear regression will be employed to examine the relationship between HEI and the outcome variables (WHOQOL-BREF, PEICS, and PSPICS scores), enabling predictive statements about the degree to which health equity is integrated in NS health service and delivery systems. Team members with expertise in epidemiology will guide the intersectional statistical analysis strategies we will use to explore the influence of various factors; multi-level modelling [106, 107], analysis of individual heterogeneity and discriminatory accuracy [108, 109], and other strategies identified in a rapid review on intersectionality in quantitative research will be used [110]. McCall’s [111] strategies for managing intersectional complexity will also be used to create different subgroupings, then multivariate regression modelling (adjusting for confounding variables) will be used to analyze whether significant differences exist in WHOQOL-BREF, PEICS and PSPICS scores across health equity indicators. If the results of the regression modelling are not significant, then non-parametric equivalents will be utilized. Once these strategies have been employed, community engagement will be done to explore the accuracy of each in terms of representing members’ experiences. Direction will be taken on how to refine the analyses, interpretation of results, and their presentation. This will allow for members of equity-denied groups to contribute to the exploration of gaps in the healthcare delivery process and relate them to unmet health needs. Strengths and addressed needs will be similarly explored.

#### Interviews: data collection and analysis

Data collection and analysis will happen simultaneously by way of constant comparison methods [99]. Canada’s Quality of Life Framework informed the development of a semi-structured interview guide (health domain, healthy care systems subdomain) and will be used to explore unmet health needs and access to timely and quality health services [112]. Semi-structured interviews and focus groups (in-person or virtual) will be conducted to explore barriers and facilitators to improving the coordination of and access to people-centred, flexible, and quality health care in NS. One-on-one interviews will last for approximately 30 min, be audio recorded, and transcribed verbatim. There will also be up to 6 focus group interviews of 5 participants each (participants grouped according to shared characteristics) that will last for up to 90 min.

##### Interpretive description

A qualitative research methodology that generates knowledge for the purpose of informing clinical practice [99, 113–115], Interpretive Description will be used for qualitative data analysis to capture shared and individual dimensions of human subjectivity in relation to domains of power operating across multiple forms of oppression. As such, Intersectionality Theory [116] will complement Interpretive Description methodologically and generate a complex analysis of healthcare delivery within the NS health system. Interpretive Description supports the use of an iterative triangulation process [117] that will promote the identification of patterns in the data and directly inform service delivery through the recommendations that emerge from our findings [118].

In accordance with Interpretive Description, reflexive memo-writing will be used by interviewers to situate themselves within role and setting, and to track decision-making throughout data collection and analysis [99]. We will employ a coding scheme (initial, then focused coding) that promotes the emergence of thematic patterns, recurring ideas, and elaboration of meaning from the data [99, 100]. The health equity lens will be used to analyze qualitative data by referring to the subject positions of each participant and comparing their intersectional identities to the data. This will allow the data to be analyzed in relation to interlocking systems of privilege and oppression that sustain health inequities. This will add a layer to our constant comparison process that will help us relate health equity to gaps and unmet needs identified in the qualitative arm of the study. Member checking, negative case analysis, and theoretical sampling will be used to promote data saturation and promote qualitative trustworthiness [85, 100, 119]. If necessary, a secondary literature review will be done to situate findings within existing evidence.

#### Integration of quantitative and qualitative data

Once phase two data collection is complete, the coherence of the different data sources will be explored through follow-up community networking and phase two consensus meetings. A triangulation protocol [120] will be developed and expanded upon by soliciting feedback from members of different NS communities through community engagement. Phase two community engagement will also be led by team members who belong to the communities being engaged, informed by phase one activities, involve the sharing of preliminary findings, and serve as a form of member checking that helps us contextualize study findings. We will re-engage those from phase one community networking and additional interest holders across the province, depending on funding and community interest. Engaging different interest holder groups will test the validity, reliability, and generalizability of data [119] and enhance data triangulation. Identified gaps and strengths in the healthcare delivery process as well as met and unmet health needs will be compared, and their convergence in meaning and prominence will be assessed. Preliminary findings on the degree to which health equity is integrated in NS health service and delivery systems will be shared with communities prior to their dissemination. Knowledge exchange events will follow phase two community engagement events. Knowledge users in various roles within the health system, postsecondary education, government, and non-profit organizations will be invited to these events that are discussed in more detail in the knowledge mobilization section.

### Phase three: use and share results

#### Knowledge mobilization

The knowledge dissemination plan (see Table 2) has three primary goals: 1) bring disparities in care coordination to light, 2) inform current and future health equity-related initiatives in NS, and 3) address a gap in the literature on how to enhance methodological rigour by operationalizing Intersectionality Theory in mixed methods research. KT goals one and two will be addressed by developing a dissemination plan for the following outputs for audiences to be determined at follow-up community engagement events and a full-team consensus meeting that takes place at the end of phase two (e.g., a policy brief (policy makers/government/health systems partners) [121], infographic (general audience) [122], peer-reviewed journal articles (researchers) [123], and a final report with guidelines/recommendations on how a health equity lens can be used to better coordinate care in NS). Physical posters and the heresearch.ca website will primarily be used alongside the research team’s existing formal networks to disseminate the report. KT goal two will also be addressed by using study findings to inform the development of a sustainable model of care that can be integrated to advance health equity in NS. KT goal three will be addressed by publishing two peer-reviewed open-access journal articles. Both will include detailed methodological descriptions on how Intersectionality Theory was operationalized; the first will be quantitative (survey results) and the second will outline implications of operationalizing Intersectionality Theory in mixed methods research, such as those related to triangulation and quantitative and qualitative data integration.

**Table 2:** Knowledge mobilization plan

Target Audience	Key Messages	KM Activities	Outcomes (*dissemination*)
Project ADDING HEAT’s research team	Supporting the conduct of equitable research across research programs	***Application***: Training opportunities, designed and delivered, will be integrated into full-team meetings held during planning and KM phases of Catalyst Grant	1. Training sessions will be delivered on how relational accountability was demonstrated and an ethical, evidence-informed approach to using local knowledge was fostered within the context of the study 2. A one-page summary on the application of these principles will be created and disseminated to the team for future use in their own research programs
General public	How the integration of health equity in the NS health system can be improved	***Application***: Phase two community engagement and six knowledge exchange events	1. Produce series of five videos and share via study website: – Results – Role of community in the project – Implications: o Intersectoral collaboration o Healthcare policy reform o Scholarly/methodological 2. Infographic of findings 3. Policy brief (government)
Government			
Health system			
Post-secondary education			
Non-profit organizations			
Researchers	Methodological innovation	***Diffusion***: Two peer-reviewed journal articles, academic conference presentation, and scholarly/methodological implications video	

### Significance

This project focuses on working with communities whose members disproportionately experience health inequities to inform an integrated model of care that promotes the advancement of health equity in NS. By identifying and exploring gaps with members of equity-denied groups who are routinely underserved in the health system, this project leverages lived experiences to improve the integration of health equity in NS health service and delivery systems. The findings from this work will also contribute to the broader literature on intersectionality in mixed methods research, potentially influencing future health equity initiatives not only in NS but across Canada and beyond. Additionally, findings will inform a future project that will engage national interest holders and knowledge users within and across sectors to develop a sustainable model of integrated care using a learning health system approach. This study will focus on following up with members of equity-denied groups that we have already engaged. This will expand our knowledge-sharing network and capacity to promote community-led initiatives in NS and across Canada.

### Ethics

This study protocol has been reviewed and approved by the institutional research ethics board at Dalhousie University (Research Ethics Board (REB) File # 2023–6903). Mi’kmaw Ethics Watch is informed on this project in an ongoing manner. Nova Scotia Health has approved a request for an REB review exemption (REB File # 1031164).

## Supplementary Information

Supplementary Material 1
