# Supplementary material for: Advancing health equity in Nova Scotia by exploring gaps in healthcare delivery: a mixed methods protocol
Source: Res Health Serv Reg. 2025 Apr 24;4:4. doi: 10.1007/s43999-025-00062-4 (PMC12021763; doi:10.1007/s43999-025-00062-4)
Supplement: Supplementary file 1 — Supplementary Material 1 [file 43999_2025_62_MOESM1_ESM.docx]

**Appendix 1**

[Greeting]

This document serves to outline how your data will be used in this study, our understanding of the steps you want us to take to make sure you keep control of your data, and the steps that you want us to take to support your self-determination as a participant in this study.

The purpose of this research is to identify and explore health equity-related gaps in service delivery. This will inform more research on how to improve the integration of health equity in Nova Scotia (NS) health service and delivery systems. We will use multiple methods to achieve the study’s purpose. Our research team is comprised of individuals working with and/or belonging to equity-denied groups in NS who will use health equity indicators (e.g., age, appearance, beliefs, belonging, citizenship, disability, ethnicity, gender, race, sex, sexual orientation, and socioeconomic class) to address the following objectives: 1) create an inventory of NS-relevant health equity-related knowledge, 2) conduct a survey and interviews to examine the integration of care in NS using a context-specific health equity lens, and 3) share what we found out about health equity-related gaps in service delivery in NS to advance health equity across the province.

This document is an agreement that is being made between you and [Principal Investigator’s name] on how your interview data will be used in this study and is in addition to, not in place of, the informed consent form for individual interviews.

We understand the OCAP® principles are critical to involving Indigenous Peoples in research. We have started a plan below to work with you on how to manage your interview data. We can meet again to talk more about this, so you can control your interview data, if you agree to participate and remain a participant in this study. You can return this to us with changes, signed, or not at all. If you choose not to return it at all, then we will manage your data in the way that it is described in this document.

This is a general population study, meaning that it does not focus on Indigenous Peoples. However, because Indigenous People are a part of the general population and our research is happening in Mi’kma’ki, we must recognize the past, present, and future connection that the Mi’kmaq People have to this land. As Treaty People, we are committed to engaging Indigenous Peoples in this study in respectful ways that promote autonomy and self-determination.

If you decide to join this study, we will apply OCAP® principles in the following ways to respect your rights because you are the rightful owner of your data:

1. We will regularly ask you for input on the study and your involvement.
2. We will share your transcript with you before analyzing it, so you have the opportunity to confirm its accuracy.
3. We will obtain your approval on the interpretation of your data.
4. We will obtain your approval before using your data in our findings, including how we use it to make recommendations.
5. We will ask for your thoughts on findings related to your data.
6. If you want, you can stay involved in the study beyond your interview and we can provide options for you that you can choose from, if you wish to explore such a role.
7. You can leave the study at any time, and we will respect your decision. Your data will be permanently deleted if you decide not to participate in the study.

[Closing]

[Include signatures page]
